# Opposing Roles of Wild-type and Mutant p53 in the Process of Epithelial to Mesenchymal Transition

**DOI:** 10.3389/fmolb.2022.928399

**Published:** 2022-06-23

**Authors:** Oleg Semenov, Alexandra Daks, Olga Fedorova, Oleg Shuvalov, Nickolai A. Barlev

**Affiliations:** ^1^ Regulation of Gene Expression Laboratory, Institute of Cytology RAS, Saint-Petersburg, Russia; ^2^ Laboratory of Intracellular Signalling, Moscow Institute of Physics and Technology, Dolgoprudny, Russia; ^3^ The Group of Targeted Delivery Mechanisms of Nanosystems, Institute of Biomedical Chemistry, Moscow, Russia

**Keywords:** p53, wild-type p53, mutant p53, EMT, epithelial to mesenchymal transition, microRNA, epigenetic regulation

## Abstract

The central role of an aberrantly activated EMT program in defining the critical features of aggressive carcinomas is well documented and includes cell plasticity, metastatic dissemination, drug resistance, and cancer stem cell-like phenotypes. The p53 tumor suppressor is critical for leashing off all the features mentioned above. On the molecular level, the suppression of these effects is exerted by p53 via regulation of its target genes, whose products are involved in cell cycle, apoptosis, autophagy, DNA repair, and interactions with immune cells. Importantly, a set of specific mutations in the TP53 gene (named Gain-of-Function mutations) converts this tumor suppressor into an oncogene. In this review, we attempted to contrast different regulatory roles of wild-type and mutant p53 in the multi-faceted process of EMT.

## Introduction

The p53 tumor suppressor protein plays the role of the “guardian of the genome” mediating the DNA damage response leading to cell cycle arrest, cellular senescence, and apoptosis (Lane, 1992). The p53 protein acts as a signal cascade integrator whose transcriptional activity is aimed against cell malignisation and contributes to the maintenance of higher order supracellular structures like tissues and organs. The ability of p53 to interact with numerous protein partners makes it instrumental in various signalling cues. In response to different forms of stress, p53 promotes the expression of genes whose products control cell cycle and apoptosis. As a transcription factor, p53 binds the DNA cognate sites in the regulatory regions of its target genes, including non-coding genes (lncRNAs and microRNAs) (Fedorova et al., 2019; Parfenyev et al., 2021). p53 is subjected to various posttranslational modifications at different sites (Marouco et al., 2013). The covalently attached groups affect its tertiary structure that dictates the functional interactions of p53 with its targets. The posttranscriptional modifications include phosphorylation, acetylation, methylation, ubiquitination, sumoylation, neddylation, O-GlcNAcylation, adenosine diphosphate (ADP)-ribosylation, hydroxylation, and β-hydroxybutyrylation (Liu Y. et al., 2019). They can mediate an opposite effect on p53 functions. Thus, ubiquitination, neddylation, sumoylation and methylation of certain lysines mark p53 for inactivation or proteasomal degradation. On the contrary, phosphorylation and acetylation generally promote p53 stabilization (Marouco et al., 2013). Moreover, there is a crosstalk between post-translational modifications, and they can regulate each other’s functions in a positive or negative manner (Marouco et al., 2013; Liu Y. et al., 2019).

Because of the ability of p53 to orchestrate numerous processes, the threat of acquiring deleterious mutations in the TP53 gene is quite imminent. Indeed, being able to interact with multiple proteins, p53 is sensitive to mutations affecting its conformation. These mutations may interfere with the normal binding of the p53 protein to both DNA and/or its protein partners without affecting others. As a result, this can lead to an imbalance in the signal cascades, followed by the emergence of positive feedback loops and the misfunction of defence mechanisms against malignant transformation. Based on this, many mutations occurring in the TP53 gene transform it from a tumor suppressor into an oncogene. Several missense mutations in the DNA binding domain of TP53, which affect its structure most severely, are called Gain-Of-Function mutations and will be the subject of this review. These mutations comprise two functional groups: contact mutants (R248Q, R248W, and R273H) and conformational mutants (R175H, G245S, R249S, and R282H). Although they all affect the structure of the DNA binding domain of p53, the contact mutants in general directly affect its DNA binding ability, while conformational mutants affect the protein folding of p53 (Alvarado-Ortiz et al., 2021). Therefore, perhaps not surprisingly, TP53 mutations are observed in more than 50% of human cancers (Bykov et al., 2018).

The process of epithelial to mesenchymal transition (EMT) is a key stage in the development of malignant tumors. Cells activating an EMT program lose their apico-basal polarity and tight junctions but acquire the ability for migration, invasion, and extravasation. EMT is a complex and multi-stage process that is accompanied by sequential repression of epithelial gene expression concomitantly with activation of mesenchymal genes (Yang et al., 2020). Notably, the cell may acquire an intermediate phenotype characterized by the presence of both epithelial and mesenchymal signs (Yang et al., 2020). As a result, the individual clones spread throughout the body to new metastatic niches. In order to colonize these niches and form the metastases, cells undergo a reverse program, Mesenchymal-To-Epithelial Transition (Kalluri and Weinberg, 2009). The specific state of the cell at a certain moment is determined by a set of accumulated mutations and a combination of microenvironmental factors.

## p53 and EMT-TFs

EMT is a key developmental process that occurs in the embryonic period and provides histogenesis and organogenesis. It also participates in some essential processes in the adult period, like wound healing (Yang et al., 2020). EMT is controlled by a number of evolutionarily conservative transcription factors (EMT-TF) that include proteins of the Zeb family (Zeb1 and Zeb2), Snai family (Snai one or Snail and Snai2 or Slug), and a protein of the basic helix-loop-helix family, Twist. They act alone or in a complex with their binding partners functioning as transcriptional activators and repressors. So, they coordinate the switching of the epithelial gene expression program to the mesenchymal one.

The malignant transformation of a cell is often accompanied by the activation of certain EMT-TF. The set of accumulated mutations, the stage of tumor development and the spectrum of signals received from the microenvironment determines the dynamics and specificity of distinct EMT-TFs. Importantly, the activating and inhibitory effects of various EMT-TFs can manifest at different rates depending on the cellular context, so cells often express both epithelial and mesenchymal markers. Therefore, a cell can be in one of a number of relatively stable states, acquiring a certain combination of epithelial and mesenchymal features. Moreover, the hybrid Epithelial/Mesenchymal phenotype may indicate a dynamic state of tumor development, and act as a prognostic marker (Godin et al., 2020; Deshmukh et al., 2021). Thus, speaking about the role of p53 in EMT, we, first of all, consider its influence on EMT-TFs and on the balance between epithelial and mesenchymal gene expression.

The effect of p53 status on EMT was shown for several carcinomas, including bladder (Wang et al., 2016) and lung cancers (Pustovalova et al., 2021). Also, the prognostic value of p53 and Snail status was revealed on the biopsy samples of endometrial carcinomas (Dragomirescu et al., 2018). Low expression of p53 together with low expression of E-cadherin may act as an independent unfavorable prognostic sign in oral squamous cell carcinoma (Fan et al., 2013). For head and neck cancer cells, it was shown that the negative status of p53 contributed to increased Slug expression (Ingruber et al., 2021). The p53 and Slug interaction was also noted for the embryonic development. The formation of the cranial neural crest is accompanied by p53-dependent Slug inhibition that prevents EMT (Rinon et al., 2011). During the embryonic development of the heart, p53 also blocks Slug-dependent EMT of epicardial cells, interfering with their invasion and migration (Jackson-Weaver et al., 2020). In general, p53 can influence the activity of EMT-TF participating in the transcriptional regulation of EMT-TF coding genes, acting on the post-translational level and interacting with numerous signalling cascades involved in EMT regulation. On the other hand, the EMT-mediated effects also depend on the p53 status of a cell. Moreover, the native and mutant p53 usually act in opposite ways reflecting their tumor suppressive and oncogenic properties, respectively. In this article we consider the molecular mechanisms underlying the link between the p53 status and EMT. The list of p53 mutations contributing to EMT is shown in Table 1.

**TABLE 1:** p53 mutations promoting EMT.

Mutation	Type of Cancer	References
Double mutation L22Q/W23S	Lung cancer	Wang et al. (2009)
C135Y	Endometrial cancer	Dong et al. (2013)
V143A	Esophageal cancer	Ohashi et al. (2010)
A161S	Head and neck cancer	Smith et al. (2013)
V173L	Lung cancer	Wang et al. (2009)
R175H	Prostate cancer	Kogan-Sakin et al. (2011)
	Esophageal cancer	Ohashi et al. (2010)
	Ovarian cancer	Yang-Hartwich et al. (2019)
C176F	Head and neck cancer	Smith et al. (2013)
N247I	Lung cancer	Wang et al. (2009)
R248W	Lung cancer	Wang et al. (2009)
		Liu et al. (2019a)
	Ovarian cancer	Yang-Hartwich et al. (2019)
R273H	Lung cancer	Wang et al. (2009)
		Kim et al. (2014)
		Jung et al. (2021)
	Colorectal cancer	Alam et al. (2016)
		Ju et al. (2019)
	Ovarian cancer	Yang-Hartwich et al. (2019)
	Endometrial cancer	Dong et al. (2013)
R280K	Breast cancer	Chen and Chiu, (2015)
		Alam et al. (2017)
K381A	Colorectal cancer	Alam et al. (2016)
K382A	Colorectal cancer	Alam et al. (2016)
L383A	Colorectal cancer	Alam et al. (2016)

## Regulation of EMT by p53-dependent miRNAs

**FIGURE 1:**
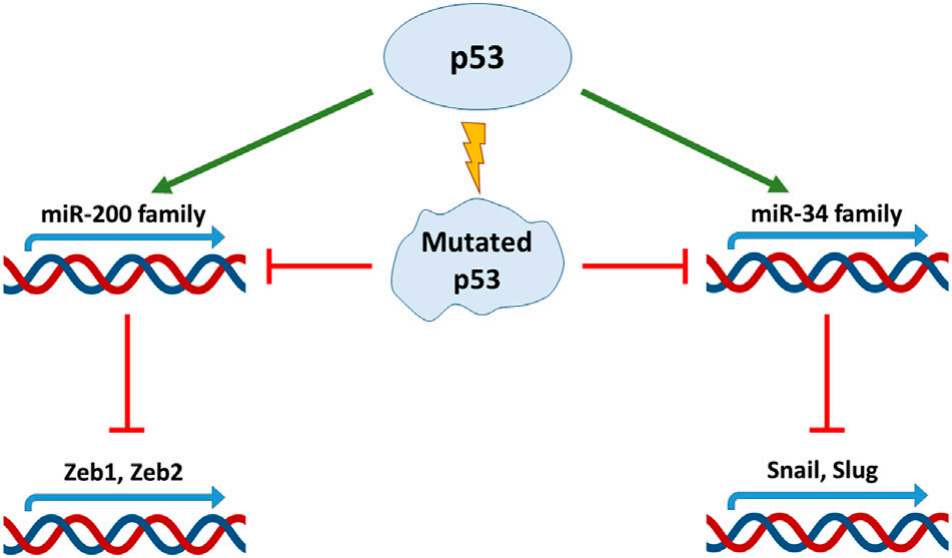
p53 regulates EMT factors through micro-RNA. The p53 protein directly regulates the expression of miR-200 and miR-34 micro-RNA families decreasing Zeb and Snail expression respectively. Mutations in TP53 gene disrupt this link and promote EMT.

Non–coding miRs are RNAs transcribed from the non-coding protein part of the genome, with a length less than 200 base pairs. They are able to complementarily bind 3′-untranslated regions of transcripts of various genes, preventing their expression and contributing to the degradation of mRNA. miRs play an important role both in the regulation of embryonic development and in the process of cell malignisation (O'Brien et al., 2018). Depending on the target, miRs can either exert tumor suppressive or oncogenic functions in the cell. They can negatively regulate the expression of proapoptotic proteins, or oppositely, enhance cell death in response to cytotoxic treatment (Lerner et al., 2012). Participation of p53 in the regulation of miRs and EMT-TF has been reported in numerous studies, e.g. for review see (Parfenyev et al., 2021). The miR-200 family members negatively regulate Zeb1 (Hurteau et al., 2007; Gregory et al., 2008), and the miR-192 family members attenuate both Zeb1 and Zeb2 (Kim T. et al., 2011). It was shown that the miR-200 family is transcriptionally regulated by p53 (Tamura et al., 2015). The data obtained from clinical samples of hepatocellular carcinoma and colorectal cancer showed that the presence of wild-type p53 increased the expression of miR-200 and miR-192 families (Kim T. et al., 2011). As a result, EMT was inhibited in p53-positive cells. On the contrary, ectopic attenuation of these miRs circumvented the effect of p53 presence in the cells (Kim T. et al., 2011; Schubert and Brabletz, 2011) (Figure 1). Additional data were obtained using MCF-7 breast cancer cells which belong to the luminal type A subtype and are characterized by wild-type p53 expression. Ectopic expression of the mutant p53 construct decreased the expression of the miR-200 family members. Importantly, attenuation or ablation of miR-200c correlated with E-cadherin decreased expression, but inversely correlated with the increased expression of Zeb1, Snail, Moesin, and Vimentin. Also, these changes in the expression of EMT markers were associated with an increased resistance of MCF-7 cells to doxorubicin (Alam et al., 2017). Notably, the effects of the p53-miR-200 axis on EMT are observed not only in cancer cells. For example, the loss of p53 expression with a corresponding decrease in miR-200 expression leads to Zeb1/Snail-mediated EMT in conjunctival cells. This event plays a key role in the pathogenesis of pterygium (Wu et al., 2016).

Transcription factors Zeb1 and Zeb2 are regulated not only by the miR-200 family members but also by other miRs. For example, it was shown that p53-dependent expression of miR-30 negatively affected Zeb2 and EMT in breast cancer cells. Ablation of miR-30 expression cancelled the effect of p53 (Di Gennaro et al., 2018). Wild-type p53 exhibited similar effects on Zeb2 through activation of miR-145 expression in hepatic stellate cells (Yang et al., 2019).

Another important family of miRs that regulates EMT is the miR-34 family. Using a colorectal cancer cell line, HCT116, and its derivative with different status of p53, it was shown p53-dependent expression of miR-34a/b/c was able to down-regulate expression of Snail, which subsequently prevented EMT (Figure 1). In line with this observation was the finding that the suppression of miR-34a/b/c was a prerequisite for TGF-β-mediated EMT in this cell type (Siemens et al., 2011). It has to be noted that in HCT116 cells, the Wnt-β catenin-LEF1 pathway is constantly activated. As a consequence of this activation, GSK3β kinase mediates phosphorylation of Snail leading to its subsequent ubiquitinylation and degradation in the proteasome. The untranslated regions of Wnt-β catenin and LEF1 contain miR- 34a/b/c binding sites. Since the expression of miR- 34a/b/c is p53-dependent, loss of p53 in these cells not only activates Snail expression, but also stabilizes it at the posttranslational level (Kim N. H. et al., 2011). Also, in colorectal cancer cells, p53 was shown to negatively regulate cytokine Interleukin six receptor (IL6-R) expression through miR-34. When miR-34 expression is suppressed, IL-6 binds to IL-6R and activates expression of Snail, Slug and Zeb1 via the STAT3 signalling pathway. The signal transducer and activator of transcription 3 (STAT3) belongs to the family of STATs (1, 2, 3, 4, 5a, 5b, and 6), which convey signals from cytokine and growth factor receptors to the nucleus where they exert their functions as transcriptional regulators. Activated by IL-6, STAT3 is able to negatively regulate miR-34 expression, thus creating a negative feedback loop (Rokavec et al., 2014). A similar effect has been detected upon HPV-induced suppression of p53 in several cancers, which resulted in the attenuation of miR-34 expression. Consequently, once the inhibitory effect of miR-34 on Bcl2, Cyclin D and Snail is removed, transformed cells increased their survival, proliferation and EMT (Chen and Zhao, 2015).

Mutations in the p53 protein radically change the nature of its miR-mediated effects on EMT. In this respect, it was shown that HCT116 cells with ectopic expression of p53-R273H released exosomes containing oncogenic miR-21-3p and miR-769-3p that target SMAD7, a powerful inhibitor of the TGF-β pathway. In addition, these miRs were elevated in clinical samples carrying mutant p53 compared to samples carrying wild-type p53. Also, fibroblasts, treated by vesicles containing these miRs, upregulated the expression of TGF-β, which, as mentioned earlier, is able to induce EMT in wild-type p53 cells (Ju et al., 2019). In endometrial cancer cells, mutant p53 (GOF: R273H, R175H and C135Y) was also shown to inhibit transcription of miR 130b, a negative regulator of Zeb1 (Dong et al., 2013). In addition, esophageal carcinoma cells carrying mutant p53 (R175H) instead of the wild-type failed to induce miR-200, -205, and miR-141. Attenuation of these tumor-suppressive miRs allowed the cells to avoid aging and activate the EMT program via the TGF-β-MOD-Eb1/Eb2 signalling pathway (Ohashi et al., 2010).

One of the mechanisms by which p53 affects miR is mediated via its interaction with DEAD-box RNA helicase p68, which is a part of the Drosha/p68 complex that is necessary for processing of miRs. Unlike the wild-type, mutant forms of p53 were shown to interfer with the normal operation of Drosha/p68 (Suzuki et al., 2009). In this respect, mutant p53 was shown to interfere with the formation of the Drosha/p68 complex in ovarian cancer cells thereby hindering the process of miR- 26a-1 maturation. This tumor suppressive microRNA targets the transcriptional repressors, EZH2 (Enhancer of Zeste Homolog 2, the component of polycomb repressive complex 2 (PRC2)) and Snail, thereby preventing EMT. Thus, mutant p53, by suppressing miR-26a-1, attenuated EMT in ovarian cancer cells (Jiang et al., 2015).

In general, the network of miRNAs, as the fundamental mechanism of gene expression regulation, is widely involved in the phenotypic plasticity of cancer cells. miRNAs-regulated EMT was observed for a variety of cancer cells. Thus, a better understanding of the signalling cues that regulate the expression of miRNAs that affect EMT can be useful from the therapeutic point of view, especially for p53-negative cases.

## p53-Mediated Effects on EMT at the Protein Stability Level

As mentioned earlier, the ubiquitin ligase Mdm2 is one of the key negative regulators of p53 at the posttranslational level. Formation of the Mdm2-p53 complex leads to polyubiquitination and proteasomal degradation of the latter. Not surprisingly, small molecule inhibitors of the p53-Mdm2 interaction are being actively investigated and some of them have reached different stages of clinical trials as anti-cancer therapeutics for different types of cancer (Davidovich et al., 2015; Lezina et al., 2015; Konopleva et al., 2020). However, p53 can also function as a scaffold, forming multi-protein complexes with Mdm2, and hence mediating Mdm2-related proteasomal degradation of its binding partners. A large number of processes, including EMT, are regulated by this mechanism (Kung et al., 2000; Brat et al., 2003).

**FIGURE 2:**
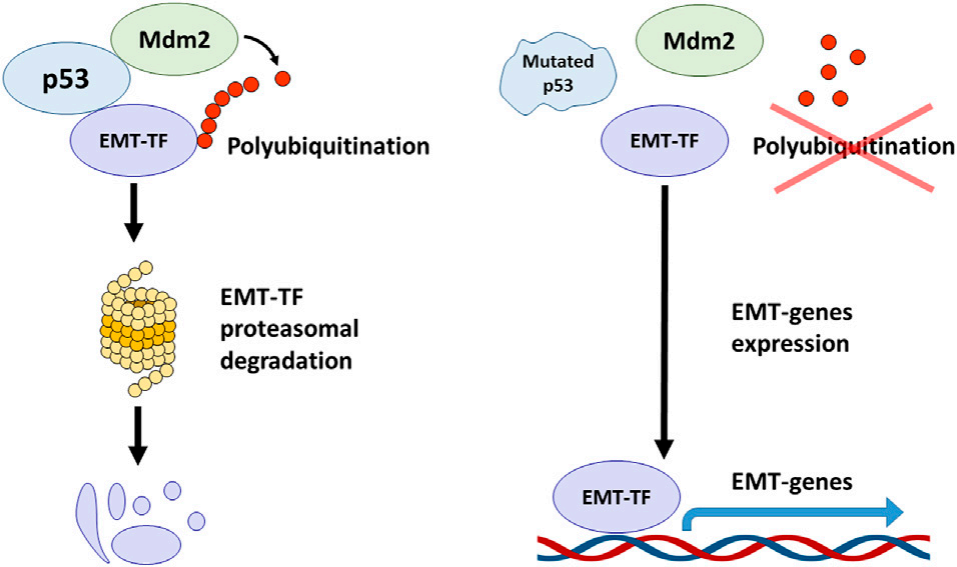
The crosstalk between p53, Mdm2 and EMT transcription factors. The wild-type p53 forms the complex with Mdm2 and EMT-TFs mediating their polyubiquitination and proteasomal degradation. TP53 mutations abolish the ability of p53 to associate with EMT-TFs and to form the complex with Mdm2 that results in EMT-TFs stabilization and promotion of EMT process.

For example, in non-small cell lung cancer lines, p53 was shown to interfere with EMT by causing the Mdm2-mediated degradation of one of the key EMT-TF: Slug (Snai2). At the same time, p53 R248W mutation prevented the formation of a complex between Mdm2 and Slug, which led to the accumulation of the latter and the onset of EMT (Figure 2). Also, similar effects were observed in breast cancer cells, MDA-MB-231 (p53 R273H) and colorectal cancer cells, SW620 (p53 R273H) (Wang et al., 2009).

On the contrary, overexpression of wild-type p53 and Mdm2 in breast cancer cells, MCF-7, led to the suppression of Slug, and subsequently increased the expression of E-cadherin and decreased invasiveness (Figure 2) (Wang et al., 2009). Using lung cancer cells, it was reported that the p21/WAF1/CIP cell cycle inhibitor, which is one of the major transcription targets of p53, is required to ensure the proteasomal degradation of Slug. The p53-p21-Mdm2-Slug quaternal complex formation is necessary for this process. It was shown that mutation R273H in p53, which suppressed its transactivation activity towards p21, also deprived p53 of the ability to reduce Slug levels. However, forced overexpression of p21 in p53 mutant cells phenocopied the effect of wild-type p53 (Kim et al., 2014).

Similar to ubiquitination, neddylation also destabilizes p53, thus preventing Slug degradation through the p53wt-p21-Mdm2-Slug complex. Thus, suppression of neddylation can decrease the migration of wild-type p53 cells via Slug degradation, but has no effect on cells with mutated p53 (Kim Y. et al., 2021). It is important to note that Slug itself can cause Mdm2-mediated proteasomal degradation of p53. It was reported that Slug promotes degradation of p53, and this required the formation of a complex with p21. The latter can also undergo Mdm2-mediated degradation, but this process is p53-independent. In addition, Slug has been shown to directly activate Mdm2 expression (Kim J. et al., 2021). Importantly, the RING domain of Mdm2 was revealed to be able to interact with RNA. In the absence of p53, instead of forming the p53-Mdm2-Slug complex that ensures the degradation of Slug, Mdm2 can stabilize mRNA of Slug via its RING domain, thus contributing to the accumulation of this protein. This process is often observed in metastases, but not in the primary tumors (Jung et al., 2013).

Interestingly (Lim et al., 2010), showed that p53 can interact in a similar way with Snail (Snai 1). Using hepatocellular carcinoma cells, the authors uncovered the effect of p53 on Mdm2-mediated proteasomal degradation of Snail, thereby reducing the ability of cells to invade. The presence of mutations in the DNA-binding domain hindered this effect. In contrast to the previous report (Cheng et al., 2021), reported that the inhibition of the p53-Mdm2 interaction, on the contrary, reduces the level of Snail in lung cancer cells. These contradictory results require further investigation.

Using preneoplastic breast cells, it was revealed that Snail can form a ternary complex with p53 and HDAC1. Its formation led to the deacetylation of p53 and subsequent degradation in the proteasomal (Ni et al., 2016). Unexpectedly, CBP/p300-mediated acetylation was found to be required for stabilization of Snail itself. Suppression of the CBP/p300-Snail complex formation led to the proteasomal degradation of Snail and the activation of p53 (Li et al., 2020). The data obtained using lung mesothelioma also showed that blocking the Snail-p53 association with a small molecule, GN25, led to p53 activation (Cho et al., 2015). Also, inhibition of the Snail-p53 axis by GN25 in A549 cells changed the expression profile of genes associated with immunoregulation, increased the expression of IL8, CXCL2, and CD81 (Hammoudeh et al., 2020). Inhibition of p53 through the interaction with Snail occurs in tumors that contain K-Ras mutations. ATM and Rad3-related proteins stabilize Snail in such K-Ras mutant cells, thereby blocking the effect of wild type (Lee et al., 2009).

Another EMT transcription factor, Twist1, also interacts with p53. Twist one interacts with the C-terminal domain of p53 in sarcoma cells, preventing its phosphorylation by Ser392. A lack of phosphorylation in the C-terminus increases the affinity of Mdm2 to p53, leading to its subsequent proteasomal degradation (Piccinin et al., 2012). It should be noted that Twist can inactivate p53 not only through ubiquitination and subsequent proteasomal degradation but also via direct association with the DNA binding domain of p53 thereby blunting its transcriptional activity. Accordingly, induction of Twist expression in prostate and breast cancer cell lines (PC-3 and MCF-7, respectively) decreased the p53-dependent expression of p21. Also, Twist1, undergoing Ser42 phosphorylation by protein kinase B (Akt), was shown to inhibit p53 activity in response to DNA damage (Vichalkovski et al., 2010).

Reciprocally, the interaction of p53 with the N-terminus of Twist prevented its DNA binding to the promoter region of the YB-1 gene, which is the target of Twist, thus inhibiting its transcription (Shiota et al., 2008). Furthermore, wild type p53 itself can direct Twist for proteasomal degradation using an E3-ubiquitin ligase, Pirh2, which itself is a target of p53. On the contrary, GOF mutations in p53 such as R175H, R248Q and R273H prevented the p53-Pirh2-Twist complex formation and stabilized the latter (Daks et al., 2013; Yang-Hartwich et al., 2019; Daks et al., 2021)

Thus, ubiquitin-dependent proteasomal degradation of the key EMT-TFs can play a decisive role in regulation of EMT. The p53 protein, by serving a scaffold for protein-protein interactions and regulating the expression of several E3 ubiquitin ligases, can function as an integrating element of this regulatory network. Thus, the level of its expression, as well as the presence or absence of mutations can affect EMT.

## Effects on EMT *via* Signalling Pathways Regulated by Mutant p53

Tumor suppressor p53 can indirectly regulate EMT by participating in various intracellular signalling cascades. Thus, the status of p53 in the cell will largely determine the phenotypic changes associated with EMT. Often, p53 serves as a link between EMT and the acquisition of resistance to genotoxic drugs, inhibition of apoptosis, and changes in metabolism. p53 participates in the coupling of EMT and signalling pathways coming from growth factor receptors, and also provides epigenetic regulation of EMT-TFs expression. Here we review a number of p53-mediated mechanisms regulating various signalling pathways during the EMT.

**FIGURE 3:**
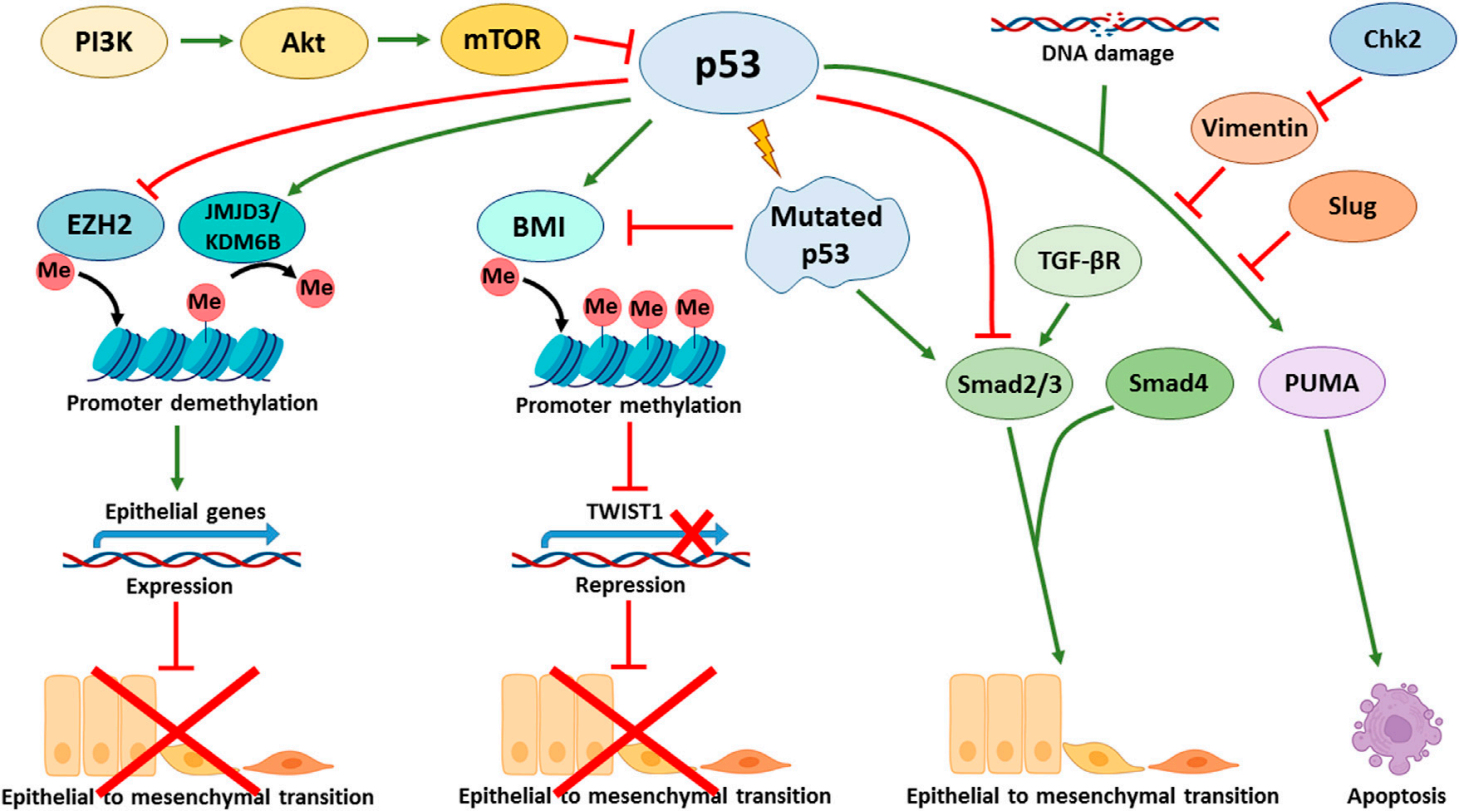
p53 affects various signalling pathways involved in the regulation of EMT. p53 directly activates the transcription of CDH1 (E-cadherin coding gene) by binding to its enhancer promoting epithelial phenotype. Additionally, p53 activates the expression of E-cadherin at the epigenetic level through recruiting JMJD3/KDM6B demethylase that removes repressive marks from histones and promotes the activation of p53 target genes. Furthermore, p53 negatively regulates EZH2 (Enhancer of Zeste Homolog 2) that represses E-cadherin. p53-mediated expression of BMI1 contributes to the maintenance of a repressive mark in the TWIST1 promoter region. Mutations in p53 lead to suppression of BMI1 and positive regulation of TWIST1. In addition, mutant p53 activates EMT via the TGF-β signalling cascade. In turn, Slug and Vimentin inhibit the p53 effects, e.g., suppressing PUMA-dependent apoptosis in response to DNA damage. Chk2 kinase is able to block the effect of Vimentin and support the p53 effects. PI3K/Akt/mTOR signalling axis negatively regulates p53, thereby contributing to EMT in p53wt cells.

The status of p53 is critical to the ability of cells that have activated the EMT program to block apoptosis and, thereby, acquire resistance to genotoxic stress caused by both gamma radiation and cytotoxic drugs. For example, the p53 R273H mutation leads to a decreased sensitivity of colorectal cancer cells to doxorubicin, both in culture and in a mouse model. Cells with mutant p53 were able to activate the EMT program and expressed stem markers CD44v6/CD133, c-Myc, and Zeb1 (Hosain et al., 2016). In line with these findings is another report showing that the expression of mutant p53 (GOF: R175H, R273H, D281G and V143A) in colorectal carcinoma cells can simultaneously increase chemoresistance and trigger EMT via the Ephrin-B2-dependent expression of EMT-TFs: Snail and Slug. Mechanistically, in response to DNA-damaging agents, the transcriptional complex p300-mut-p53-NF-Y is formed on the promoter of the EFNB2 gene to facilitate its transcription (Alam et al., 2016). Stabilization of otherwise mutated p53 with small molecules can contribute to Mdm2-mediated degradation of Slug and cause apoptosis of endometrial cancer cells also occurring with the participation of PUMA (Figure 3) (Liu et al., 2020). On the other hand, in p53-positive colorectal cancer cells that undergo the EMT program, a protein complex between Vimentin and wild type p53 is formed that prevents the translocation of p53 into the nucleus and hence inactivates its transcriptional activity. Inactivation of p53 conferred resistance of these cells to 5′ fluorouracil. Pharmacological activation of checkpoint kinase 2 (Chk2) destroyed this complex, restored p53 activity and decreased chemoresistance (Figure 3) (Katoch et al., 2021).

The p53 tumor suppressor also participates in regulation of EMT by controlling the PI3K/Akt/mTOR signalling pathway. It was shown that pharmacological inhibition of the PI3K/Akt/mTOR axis in hepatocellular carcinoma cells increased p53 activity and attenuated EMT (Figure 3) (Li et al., 2019). Experiments conducted on non-small cell lung cancer lines demonstrated that blocking the AXL tyrosine kinase receptor also reduced EMT by suppressing the PI3K/Akt/mTOR signalling, leading to an increase in p53 levels. The latter, in turn, increased the sensitivity of cells to EGFR inhibitors (Suresh et al., 2019). Similarly, in gastric cancer cells, a decrease in EMT was demonstrated through the inhibition of PI3K/Akt/mTOR by activation of p53 (Xu et al., 2020). On the other hand, activation of the PI3K/AKT/mTOR signalling by Twist in breast cancer cells leads to inhibition of p53 at the transcriptional level and changes in cellular metabolism (Yang et al., 2015).

The TGF-β signalling pathway is one of the major EMT inducers, is also affected by p53. Thus, in hepatocellular carcinoma cells treated with TGF-β, a higher level of EMT activation was reported in p53 knockdown cells versus control cells (Wang et al., 2013). In support of these results are the data obtained on mouse mammary gland epithelial cells: one of the p53 inactivating GOF mutations, R277C, blunts transcriptional activity of p53 and thereby contributed to a more intense activation of EMT under the action of TGF-β (Figure 3). Interestingly, knockdown of p53 phenocopied this result. Apparently, this phenomenon is solely dependent on the transcriptional activity of p53 (Termén et al., 2013). Mutant p53 R175H was shown to affect the targets of the TGF-β pathway without affecting the phosphorylation of R-Smads. Phosphorylation of this mutant p53 protein at Ser392 induced by Erk kinase, confers the ability of p53 R175R to bind the MH2 domain of Smad3, while the wild-type p53 protein primarily binds the MH1 domain of Smad2. As a result, the formation of the Smad3/Smad4 complex is disrupted. It is known that expression of Slug and MMPs depends on Smad2. Therefore, blocking of Smad3, which is a binding partner of Smad2, prevents the activation of Smad-mediated pathway, possibly eliminating the negative regulation of EMT (Ji et al., 2015). Also, mutant p53 (R175H) in the lung and ovarian cancer cells can directly reduce the expression of TGF-β Receptor II, which leads to a decrease in MMPs expression (Kalo et al., 2007). Mutant p53, through inhibition of p63 expression, promotes invasion and migration of breast cancer cells upon activation of TGF-β and Ras-CK1 pathways. Smad2 promotes the formation of a complex with phosphorylated (upon activation of Ras-CK1) mutant p53 and p63, which interferes with the functions of the latter (Adorno et al., 2009).

Participation of p53 in many other signalling pathways influencing the onset of EMT was reported. For example, wild-type p53 was shown to inhibit K-RAS activity and reduce Erk-mediated EMT in the mammary gland epithelium cells (Zhang et al., 2013). Data obtained on squamous carcinoma cells of head and neck cancers indicates that in cells with mutant p53 (loss of function of C176F and A161S), silencing of the p65 subunit of the NF-kB complex leads to activation of EMT, while on the contrary, in cells with wild-type p53 overexpression of NF-kB activates EMT (Lin et al., 2015). Experiments on esophageal carcinoma cells showed that R172H mutation in the p53 protein reduced the expression of Rab11-FIP1. This protein is a partner of Rab11 and Rab25, which ensures the recycling of receptors from endosomes to the plasma membrane. However, the loss of its expression activated EMT along the Zeb1-mediated transcriptional program (Tang et al., 2021). In a mouse model of ovarian cancer, it was demonstrated that wild-type p53 induced phosphatase 1 (Wip 1) blocks EMT by affecting the activity of ATM kinase. Activated ATM is able to phosphorylate Akt, which leads to suppression of GSK-3ß and hence stabilization of Snail. Thus, Wip1 is able to suppress EMT by indirectly reducing the activity of Snail (Yin et al., 2016). In K-Ras mutant cell lines, ATR kinase, not ATM, was found to play the key role in stabilizing Snail via suppression of GSK-3ß activity and blocking p53 (Lee and Park, 2011). Furthermore, Snail, which is regulated by the HGF/c-Met signalling axis, was shown to control the invasive potential of hepatic stellate cells. At the same time, the loss of p53 removes its inhibitory effect on c-Met expression, which enhances the metastatic effect of this signalling pathway (Liu et al., 2016). On the other hand, the opposite effect of wild-type p53 was demonstrated on hepatocellular carcinoma cells. p53 positively regulated the expression of Notch1 at the transcriptional level, which subsequently enhanced the invasive potential of cells with increased Snail expression (Lim et al., 2011). Slug activity also depends on various signalling pathways that involves p53. Results obtained on non-small cell lung cancer cells and cervical cancer cells showed that mutations in p53 prevented the activation of DDX3 (DEAD box RNA helicases) expression, which, together with SP1, are responsible for Mdm2 expression. A decrease in Mdm2 expression and its association with mutant p53 contributed to Slug stabilization and subsequent EMT activation (Wu et al., 2014). The L1 cell adhesion molecule (L1CAM) promotes invasion of NSCLC cells. Slug and beta-catenin activate its expression, while wild-type p53, on the contrary, suppresses it (Liu X. et al., 2019).

In general, aberrant activation of pro-oncogenic signalling pathways leads to the formation of self-sustaining positive feedback loops, which eventually leads to cell malignancy. Clones with the capacity for phenotypic plasticity acquire a selective advantage and contribute to further development of a tumor. The p53 tumor suppressor acting as the main nodal element of the cellular defense system against malignant transformation plays role in the maintenance of a stable epithelial phenotype by preventing phenotypic plasticity via negative regulation of EMT-TFs. The opposite is also true, i.e. activation of the EMT-program requires down-regulation of p53. Collectively, p53 counteracts EMT via different mechanisms depending on the cellular context. Conversely, GOF mutations in the p53 protein significantly enhance the metastatic potential of tumor cells.

## Epigenetic Regulation of EMT

p53 can also regulate EMT at the level of epigenetics. We, and others, have shown that p53 can target various histone methyltransferases and acetyltransferases to the regulatory regions of its target genes (Marouco et al., 2013; Zhu et al., 2015; Rada et al., 2017). It was shown that p53 can interact with JMJD3/KDM6B demethylase (Figure 3), which removes the repressive epigenetic marks, H3K27me3 and H3K27me2 from histones H3. In this way, genes bearing p53 binding sites can be activated (Williams et al., 2014). At the same time, wild-type p53 can promote the opposite epigenetic activity, i.e. H3K27 methylation. However, this seems to be an indirect effect, because it is mediated via p53-dependent expression of BMI1, which establishes the repressive methylation mark, H3K27me3, in the promoter region of TWIST1 gene (Figure 3). Importantly, the presence of mutations in the p53 protein (e. g. p53R175H) prevents the repression of TWIST1 transcription (Figure 3). (Kogan-Sakin et al., 2011). Thus, the p53 mutation status may contribute to the activation of Twist expression in prostate epithelial cells.

Moreover, p53 can regulate the expression of epithelial genes like CDH1 (encodes for E-cadherin) directly, without the involvement of EMT-TF. To do so, p53 directly binds to the CDH1 enhancer and facilitates transcriptional activation. On the other hand, p53 is involved in the mechanism of epigenetic regulation, in which it suppresses the activity of EZH2 (Figure 3), preventing the appearance of the repressing H3K27-3me mark in the promoter region of CDH1 and hence preserving the activating H3K27-ac covalent modification. Apparently, these effects are cell context dependent. Various cells may differ in sensitivity to the effects conferred by p53. For example, in lung cancer cell line A549, p53-dependent acetylation of histones at the regulatory regions of CDH1 is necessary for its efficient transcription. On the contrary, breast cancer luminal cells, MCF-7, were shown to be insensitive to the status of p53 and demonstrated the absence of histone acetylation in the regulatory region of CDH1. Despite the lack of acetylation, the expression of E-cadherin is preserved and the cells do not undergo EMT (Oikawa et al., 2018).

During carcinogenesis, epigenetic regulation, which controls gene expression, undergoes significant changes. This makes the process of epigenetic regulation in cancer cells an attractive target for pharmacological intervention as a novel anti-cancer therapeutic strategy. Since EMT is also regulated on the level of epigenetics, it would be interesting to see whether inhibitors of chromatin-modifying enzymes also block EMT.

## Conclusion

EMT can be envisioned as a process of cell transition from one stable state to another, which is accompanied by the rewiring of a large number of signalling cascades, and the acquisition of new phenotypic properties. A wealth of data strongly suggests that p53 regulates EMT at various levels. p53 stabilizes the epithelial phenotype by interacting with chromatin remodelling proteins, thereby contributing to the repression of EMT-TF genes and to the activation of epithelial gene expression. p53 also regulates EMT at the transcriptional and post-transcriptional levels through miRs and long non-coding RNAs and direct interactions with TFs. Furthermore, p53 regulates EMT-TF at the posttranslational level through the ubiquitin-proteasomal degradation system. Another poorly explored area controlled by p53 is the metabolic reprogramming of cancer cells during EMT (McDonald et al., 2017; Shuvalov et al., 2021). The fact that p53 can change its cellular functions depending on the acquisition of missense mutations makes the mutant p53 protein even more important regulator of EMT. For example, mutant p53 was shown to facilitate the production of exosomes, suggesting that mutant p53 may regulate the microenvironment of metastatic niches (Pavlakis et al., 2020). Furthermore, since metastasizing is a multi-step process that involves EMT, intravasation into the blood vessel followed by extravasation and Mesenchymal-to-Epithelial Transition (a process, opposite to EMT), it would be interesting to see whether mutant p53 can affect all these steps (Giacomelli et al., 2018). For, example, to switch from the dormant state to proliferative state, cancer cells have to reprogram their metabolism. In this respect, p53 is known to affect mitochondria to promote the Warburg effect in cancer cells (Matoba et al., 2006). Finally, an important question is how the mutant form affects the immune surveillance which prevents the uncontrolled spreading of metastases.
